# Microstructures of a SiC–ZrC Ceramic Fiber Derived from a Polymeric Precursor

**DOI:** 10.3390/ma13092142

**Published:** 2020-05-06

**Authors:** Min Ge, Xiaoxu Lv, Hao Zhang, Shouquan Yu, Zhenxi Lu, Weigang Zhang

**Affiliations:** 1 State Key Laboratory of Multi-phase Complex Systems, Institute of Process Engineering, Chinese Academy of Sciences, Beijing 100190, China; gemin@ipe.ac.cn (M.G.); zhanghao715@mails.ucas.edu.cn (H.Z.); sqyu@ipe.ac.cn (S.Y.); zhxlu@ipe.ac.cn (Z.L.); 2 National Key Laboratory of Advanced Composites, Beijing Institute of Aeronautical Materials, Aero Engine Corporation of China, Beijing 100095, China; xiaoxul@126.com; 3 School of Chemical Engineering, University of Chinese Academy of Sciences, Beijing 100049, China

**Keywords:** ceramic fiber, silicon carbide, zirconium carbide

## Abstract

Continuous ceramic fiber comprising silicon carbide–zirconium carbide (SiC–ZrC) binary phases was obtained through melt spinning, electron-beam curing and pyrolysis of a pre-ceramic precursor of polyzirconocenecarbosilanes (PZCS). After pyrolysis and heat treatment, ZrC particles with mean diameters of 15–20 nm were formed and homogeneously dispersed in a matrix of fine crystalline β-SiC with an average grain size of 6–10 nm. Concentration of Zr in the fiber varies from 14.88% to 17.45% by mass. Fibers consisting of near-stoichiometric ZrC and SiC with little free carbon can be obtained through pyrolysis decarbonization of the as-cured fiber in hydrogen from room temperature to 1000 °C, and subsequently heat treatment in argon up to 1600 °C for 1 h. High-temperature treatment of these amorphous inorganic fibers leads to crystallization of the binary phases of β-SiC and ZrC. The removal of free carbon under hydrogen results in more rapid growth of β-SiC and ZrC crystals, in which obvious aggregation of the dispersed ZrC particles among the continuous β-SiC matrix can be ascribed to a fast migration of Zr cation.

## 1. Introduction

Silicon carbide (SiC) fiber synthesized from polycarbosilane is one of the most important reinforcements for ceramic matrix composites (CMCs), which are now finding more and more applications to meet harsh environments of high temperature and air-oxidation such as turbo-engine blades in aerospace industry [1,2,3,4,5]. Polycrystalline SiC fiber exhibits brittle fracture behavior at room temperature but being ductile under applied certain stress at temperatures above 1200 °C. In fact, plastic deformation and rupture caused by creep has become a key limitation of this material for any possible long-time applications at temperatures above 1200 °C under loading [6,7,8,9].

In general, SiC does not melt at any known temperature and its high decomposition temperature (approximately 2700 °C) makes it natural candidates for high temperature applications without the risk of creep failure under temperatures of 1200 °C (~0.5T_m_, in K) [10,11]. However, a recent research showed that a cavitation-governed creep of crystalline SiC fine fibers with diameters smaller than 15 microns occurs dramatically at 1200 °C [5]. Amorphous silica (glass phase) and crystalline oxides (alumina or titanium oxide) with low melting points existing along grain boundaries (GBs) of SiC fine grains enhance creeping. Therefore, larger grain size in stoichiometric SiC fibers leads to both, minimum numbers and high viscosity of GBs, which results into suppressing cavitation movement and GBs sliding. On the other hand, the larger crystalline size of SiC in a continuous fiber results in extremely high modulus (about 440 GPa for H-Nicalon type S) with decreased tensile strength and toughness. Thus, the rigid SiC fibers increase difficulties in weaving quite as well as a rise of the cost caused by purification and growth of SiC grains at higher temperatures and extended retention time [12,13].

Crystallization and strengthening of GBs in polycrystalline SiC can also be achieved via precipitation or introduction of non-soluble secondary phase with a higher melting point and modulus than SiC. Creep in SiC fiber can be retarded by introduction TiB_2_ with only 2.4% in mass. It was found that the incorporated ~50 nm TiB_2_ particles reside in triple point of SiC GBs which limits the sliding of SiC [14,15]. Thus, some high-melting carbides and borides such as zirconium and hafnium are an essential prerequisite for using as resistance to creep in SiC fiber. With this attempt, direct polymerization of 1-methylsilene into polycarbosilanes has been investigated using various metallocenes as catalyst during surface dechlorination of dichloromethylsilanes by sodium [16]. For the first time, we have shown a metallocene catalytic insertion polymerization of tautomeric 1-silene into polycarbosilanes as analogs of polyolefins [16,17]. The polycarbosilanes synthesized through this molecular insertion process is suitable for spinning into SiC–ZrC composite ceramic fibers. These transition metal carbides may act as reinforcements that improve the creep resistance as well as the thermal and oxidation resistance of the SiC ceramic [18].

## 2. Materials and Methods

### 2.1. Polymeric Precursors

Polyzirconocenecarbosilane (PZCS) was synthesized from dimethyldichlorosilane, zirconocene dichloride and metallic sodium in toluene and used as precursor for the fabrication of the SiC–ZrC composite fiber. The synthesis procedure and pyrolysis behavior of PZCS polymer have been reported in detail [16,17], which was a product of zirconocene catalytic insertion polymerization of 1-methylsilene transient intermediates (CH_2_=SiHCH_3_) with a molecular Equation (1) [13], herein R = CH_3_ and n = 10–25. The polymer has an average molecular weight of 1080 g/mol, as determined by a gel-permeation chromatography (GPC) using toluene as the eluent and polystyrene as the calibration standard. The softening point of PZCS for melt spinning is around 120 °C and the ceramic yield in Ar at 1000 °C is 58%.


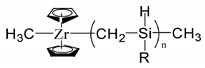
(1)

### 2.2. Fabrication of Fibers

PZCS about 40 g was charged into the spinning can and heated to the spinning temperature (135–140 °C) under a nitrogen atmosphere and then extruded through a single-hole spinneret with a diameter of 0.25 ± 0.05 mm. The PZCS green fibers were cured in a flow reactor in argon by electron-beam irradiation (beam current of 1.0–2.5 mA, retention time of 3–5 h and dose for the curing is about 5–8 MGy). The as-cured fibers were heated to 1000 °C under H_2_ or Ar atmosphere, then heated to 1600 °C under Ar atmosphere and maintained at 1600 °C for 1 h. In above-mentioned two cases, a heating and cooling rate is 2 °C/min. For ease of description, the former was marked as H_2_–Ar process fiber and the latter was marked as Ar–Ar process fiber.

### 2.3. Characterizations

The elemental contents in the fibers were analyzed, in which the contents of Si and Zr were measured by ICP-OES in a Thermo Fisher ICAP6300 spectrometer (Waltham, MA, USA), the contents of carbon and hydrogen were acquired by an Elementar Vario EL determinator (Langenselbold, Germany). The TC-436 N/O analyzer was used to determine the content of oxygen element (LECO, St. Joseph, MI, USA).

The phase compositions in the pyrolysized fibers were identified by X-ray diffraction (XRD, PANalytical X’Pert-PRO diffractometer, Eindhoven, Netherlands) at 2θ = 10°–90° with Cu K_α_ radiation (λ = 0.15406 nm at 40 kV and 30 mA).

Free carbon in the fibers was examined with a Raman micro-spectrometry (Horiba Jobin-Yvon, Paris, France), using the green line of a He-Ne laser (632.8 nm) as excitation source and scattering was measured in the first-order spectrum ranging 900–2000 cm^−1^.

The microstructures and elemental concentrations of the particles in the fibers were characterized with scanning electron microscopy (SEM, S4800, Hitachi, Tokyo, Japan) and transmission electron microscopy (TEM, TecnaiG20, FEI, Hillsboro, OR, USA) equipped with an X-ray energy dispersive spectrometer (EDS). The samples were sprayed with a carbon film and then observed with SEM.

## 3. Results and Discussion

### 3.1. Morphologies of the Polymeric and Ceramic Fibers

The used precursor PZCS is a thermoplastic polymer, which shows excellent spinnability around 150 °C, but the derived green fiber will undergo remelting and lose their fabric shape before thermosetting and pyrolysis into inorganic fiber. Therefore, curing or aging of this green fiber into thermosetting one is the first key step herding the following inorganic chemical transformation. It was well known that a traditional polycarbosilane can be cured by oxidation in hot air or oxidized gases such NO_2_, which happens via chemical reactions between Si-H with oxygen into Si-O-Si linkage and water [1,12,14]. This curing clearly occurs starting from the surface of the fiber and goes slowly into deeper site governed by oxygen diffusion. Oxidation curing will inevitably and in-homogenously introduce oxygen into the polymeric fibers, which leads to a silicon-carbon-oxygen complex formation in the organic fiber after pyrolysis. Therefore, irradiation of the fiber by electron-beams (EB) with high energy was applied for a homogeneous curing of the green fiber without introducing of oxygen contamination, which is also applied in this study. Mechanisms of this thermosetting process based on elimination reaction between two Si-H into Si-Si linkage and hydrogen releasing has been investigated and discussed by Takeda et al. [13].

**Figure 1:**
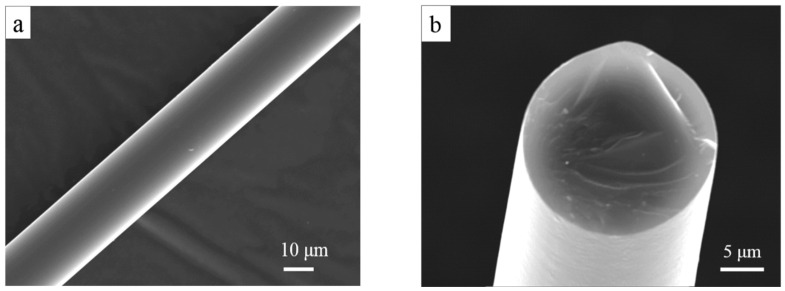

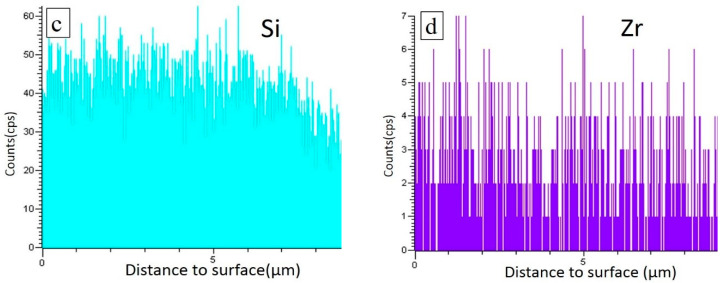
Scanning electron microscopy (SEM) images of the surface (**a**) and cross-section (**b**) of the electron-beams (EB)-cured polyzirconocenecarbosilanes (PZCS) fiber and X-ray energy dispersive spectrometer (EDS) images (**c**) and (**d**) from surface to core of the fiber.

Surface and cross-section morphologies of the EB-cured PZCS fiber are shown in Figure 1a,b, which shows a smooth surface and very dense cross-section fracture morphology of the green fiber after EB-curing in argon. The EDS images of Si and Zr distribution from the surface to core are shown in Figure 1c,d. No aggregation of zirconium phase is observed on the surface and cross-sectional parts of the as-cured fiber.

**Figure 2:**
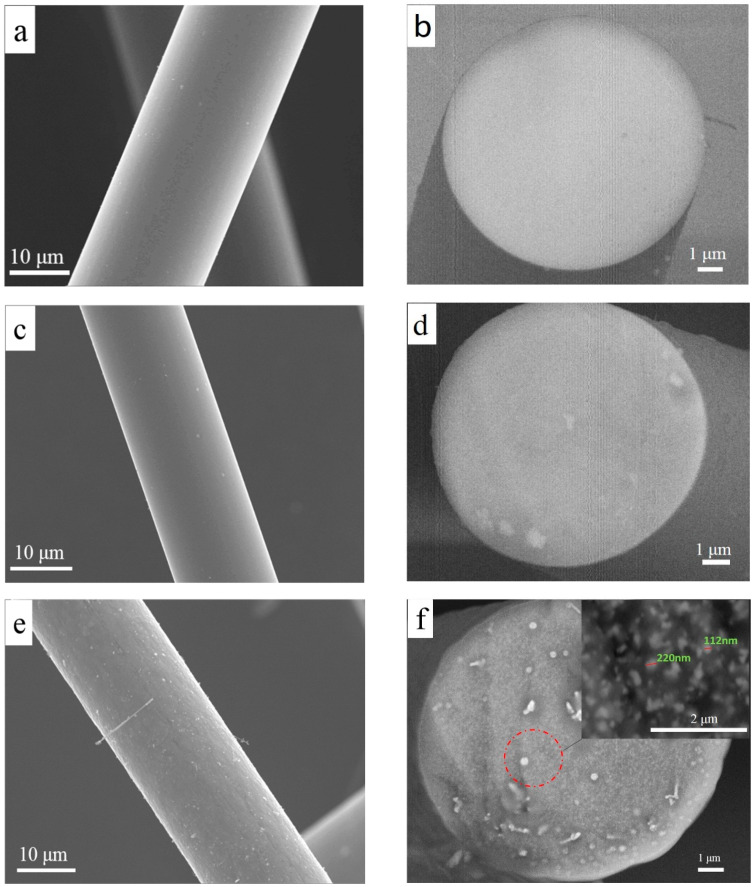
Surface and cross-sectional SEM images of the as-cured PZCS fibers after H_2_–Ar process up to various temperatures of (**a**,**b**): 1200 °C; (**c**,**d**): 1400 °C; (**e**,**f**): 1600 °C, wherein (**b**,**d**,**f**) are the backscattered electron images.

The as-cured fibers are then transferred into a thermosetting state that does not undergo remelting during pyrolysis up to 1000 °C either in H_2_ or in Ar atmosphere. Pyrolysis of the PZCS in Ar finally leads to formation of ZrC, SiC and free carbon in the residual inorganic fibers after releasing of complicated gaseous species such as methane, hydrogen and silanes [18]. The surface and cross-sectional morphologies of the ceramic fibers treated by H_2_–Ar process or Ar–Ar process at the temperatures of 1200, 1400 or 1600 °C show minor differences from each other. Figure 2 shows SEM images of the surface and cross section of the fibers obtained by H_2_–Ar process 1200, 1400 or 1600 °C for 1 h. In all three cases, the ceramic fibers show very dense and homogeneous microstructures without any visible cracks, voids or other flaws.

The backscattered electron (BSE) image mainly reflects the distribution of elements on the sample surface. The brighter the region, the higher the atomic number. BSE images of the fibers at 1200 °C (Figure 2b) and 1400 °C (Figure 2d) show a bright image, from which the SiC and ZrC in the fibers cannot be distinguished. The contrast of bright and dark regions are observed in the image at 1600 °C (Figure 2f), wherein Zr-rich brighter spots with the diameter of about 200 nm are dispersed in darker Si-rich matrix. It can be seen that obvious aggregation of Zr in the fibers is more likely to occur at 1600 °C, which may be ascribed to the faster migration of Zr cations at higher temperatures.

### 3.2. Phases Composition in the Ceramic Fibers

**Figure 3:**
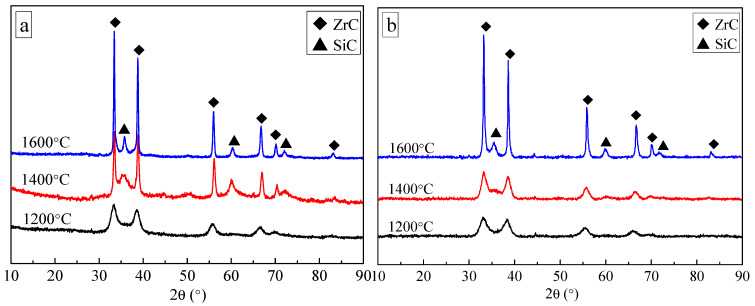
XRD patterns of SiC–ZrC ceramic fibers through (**a**) H_2_–Ar process and (**b**) Ar–Ar process up to various temperatures of 1200, 1400 or 1600 °C.

XRD analysis of the above ceramic fibers annealed by H_2_–Ar process at 1200, 1400 and 1600 °C for 1 h is shown in Figure 3a. It is indicated that ZrC is the only crystalline phase existing in the ceramic fibers after annealing at 1200 °C. With the temperatures up to 1400 and 1600 °C for 1 h, both of the crystalline phases of ZrC and SiC are identified in the ceramic fibers. The sharper diffraction peaks at 1600 °C than those at 1400 °C indicate a better crystallinity, which is in accordance with the SEM results.

XRD analysis of the other ceramic fibers by Ar–Ar process up to various temperatures of 1200, 1400 or 1600 °C is given in Figure 3b. According to the XRD patterns, the major phase existing in the ceramic fibers obtained at 1200 and 1400 °C is also only ZrC. When the annealing temperature is up to 1600 °C, both crystalline phases of ZrC and SiC can be identified in the ceramic fibers, which indicates that the crystallinity of ZrC and SiC increases with increasing temperatures.

Compared the results shown in Figure 3a,b, it is concluded that the diffraction peaks of crystalline SiC formed by the Ar–Ar process at 1200 °C are close to those appeared by the H_2_–Ar process. With the temperature up to 1400 °C or 1600 °C, the diffraction peak shapes of crystalline ZrC formed via the H_2_–Ar process become sharper than those formed via the Ar–Ar process. It is also very clear that the crystallinity of SiC formed via the H_2_–Ar process is better than that via Ar–Ar process when the heat treatment is up to 1600 °C. That is, the introduction of H_2_ atmosphere below 1000 °C has effect on the growth of ZrC and SiC grain sizes at 1600 °C, which is got to know via the following analysis.

Table 1 lists the elemental compositions and C/(Si + Zr) Atomic ratio of different fibers. Compared with green fibers, the fibers after pyrolysis at 1000 °C in Ar or H_2_ atmosphere consist of Zr, Si, C and O elements. With the pyrolysis atmosphere changing from Ar to H_2_ below 1000 °C, the Si content increases from 43.82% to 51.95%, the Zr content from 14.88% to 17.10%, and the carbon content decreases by about 10%, which results in the decrease of the C/(Si + Zr) atomic ratio from 1.90 to 1.15. After the Ar–Ar process or H_2_–Ar process at 1600 °C, the contents Si and Zr slightly increase while the carbon content further decreases, which can be ascribed to carbothermal reduction of C and O elements. The C/(Si + Zr) atomic ratio in the fibers by H_2_–Ar process at 1600 °C is 1.11, which means the fibers consist of near-stoichiometric ZrC and SiC.

**Figure 4:**
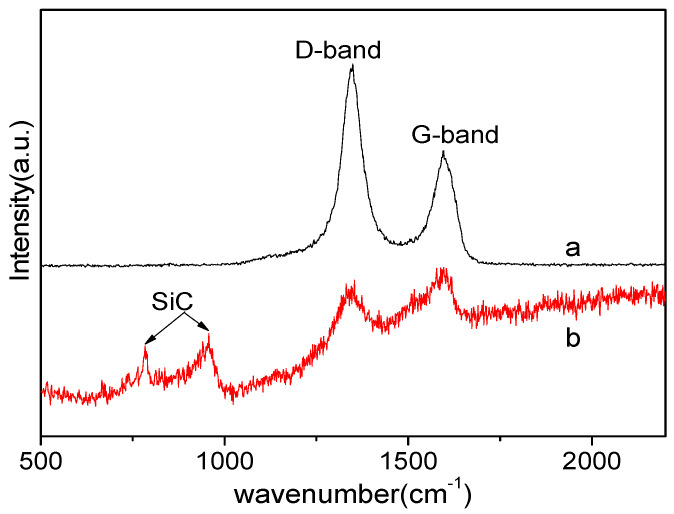
Raman spectra of the SiC–ZrC fibers obtained at 1600 °C via the Ar–Ar process (**a**) and the H_2_–Ar process (**b**).

It was known that pyrolysis of PZCS in Ar led to the formation of ZrC, SiC and free carbon in the resultant fiber [18]. Then free carbon remaining in the fibers obtained at 1600 °C is analyzed and determined by its micro-Raman spectra (Figure 4). For the fibers obtained via the Ar–Ar process, the strong and sharp peaks at 1358 and 1590 cm^−^^1^ are recorded. The scattering peak at 1590 cm^−^^1^ is ascribed to the E_2 *g*_ mode of the graphene layers and usually labeled as G band (name after “graphite”), while the scattering peak at 1358 cm^−^^1^ is designated to the D band of pyrolytic carbon (named after “defect”). The ratio of intensities of D band and G band is larger than 1, which means a large amount of free carbon exists in ceramic fiber obtained in argon at 1600 °C. In the fibers obtained via the H_2_–Ar process, the intensities of both peaks at 1358 and 1590 cm^−^^1^ become very weak, which means free carbon in the SiC–ZrC ceramic fibers is almost removed by H_2_.

Compared with the Roman spectra of the SiC–ZrC fibers obtained via the Ar–Ar process (Figure 4a), the peaks at 784 and 955 cm^−^^1^ ascribed to the β-SiC in Figure 4b are identified, which means a better crystallization of β-SiC in the ceramic fibers obtained via the H_2_–Ar process.

From the elemental analysis and Raman spectra, it is found that a larger amount of carbon can be removed from the as-cured fibers by the introduction of H_2_ atmosphere below 1000 °C. Benefiting from the decarbonization of H_2_, the production of free carbon in the ceramic fibers is reduced and the crystallinity of ZrC and SiC grain sizes is increased, as well as stoichiometric ZrC and SiC can be obtained.

**Figure 5:**
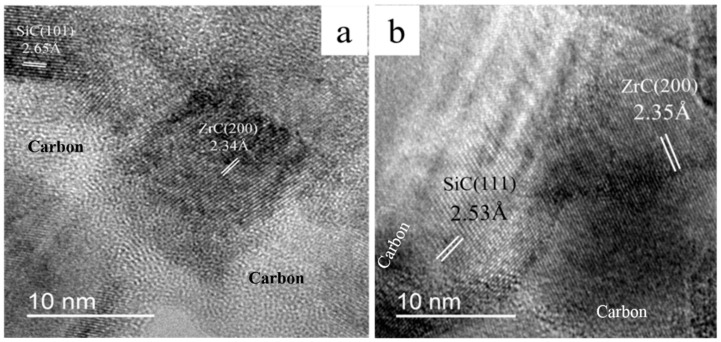
HR-TEM images of the as-cured fibers obtained via the Ar–Ar process up to (**a**) 1400 and (**b**) 1600 °C.

**Figure 6:**
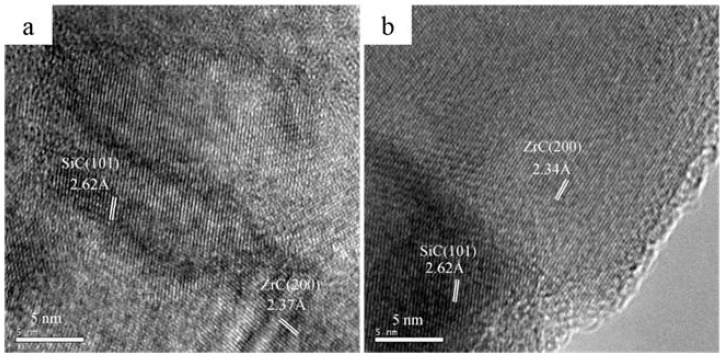
HR-TEM images of the as-cured fibers obtained via the H_2_–Ar process up to (**a**) 1400 and (**b**) 1600 °C.

Figure 5 shows high-resolution TEM (HR-TEM) images of the as-cured fibers after the Ar–Ar processes up to 1400 or 1600 °C. It can be seen that amorphous carbon is observed around ZrC and SiC nanocrystallites. In contrast, the ceramics fibers obtained via the H_2_–Ar process consist of two clearly defined phases of SiC and ZrC while free carbon is hardly observed in Figure 6a,b. These results confirm the analysis of Raman spectra.

**Figure 7:**
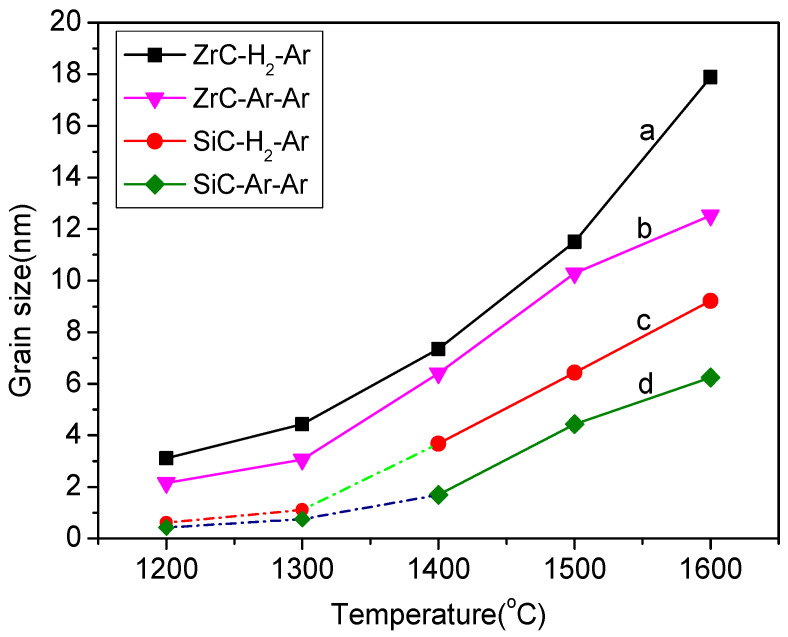
Crystalline grain sizes of ZrC (**a**,**b**) and SiC (**c**,**d**) in the composite fibers after pyrolysis at 1000 °C and annealing at various temperatures from 1200 to 1600 °C (**a**,**c**: H_2_–Ar process; **b**,**d**: Ar–Ar process).

Based on the data of X-ray powder diffraction and the Debye-Scherrer formula, the average grain sizes of SiC and ZrC in the ceramic fibers heated at various temperatures are computed, as shown in Figure 7. When the heat treatment temperature at 1200–1300 °C, ZrC crystals are formed first with the size of about 2–4 nm. With the heat treatment temperature from 1400 up to 1600 °C, the grain size of ZrC is up to 10 nm or even larger. The crystalline grain size of ZrC at 1600 °C is around 8–10 nm larger than that of SiC, which may be related to the fact that Zr cations aggregate in the fibers at higher temperatures. After heat treatment at 1600 °C via the H_2_–Ar process, the average crystalline grain size of ZrC is about 18 nm (Figure 7a) and the size of SiC is also increased to about 8 nm (Figure 7b). The crystalline grain sizes of ZrC and SiC obtained at 1600 °C via the H_2_–Ar process are 3–5 nm more than those via the Ar–Ar process. From this tendency, it is supposed that the rapid growth of ZrC and SiC crystalline grains obtained via the H_2_–Ar process will be kept and the growth of ZrC and SiC grains obtained via the Ar–Ar process will become lower.

## 4. Conclusions

A composite ceramic fiber of SiC–ZrC was fabricated from a single polymeric precursor of polyzirconocenecarbosilane, and it was shown that both of stoichiometric β-SiC and ZrC in the fibers could be formed through decarbonization of the electron-beam cured green fiber in hydrogen up to 1000 °C and subsequently annealing the inorganic fiber in argon up to 1600 °C. The microstructures of the SiC–ZrC fibers exhibited homogenously dispersion of nano-sized ZrC crystallites (~18 nm) in a matrix of β-SiC with smaller grain size (~8 nm). After pyrolysis in hydrogen below 1000 °C, a more rapid growth of ZrC and SiC crystalline grains occurred in Ar up to 1400 or 1600 °C. In the same ceramic fiber, the crystalline grain size of ZrC was larger than that of SiC and the aggregation of Zr became apparent at 1600 °C.

## Figures and Tables

**Table 1:** Content and C/(Si + Zr) Atomic ratio of different fibers.

Content (wt %)	Si	C	Zr	O	H	Cl	C/(Si + Zr) Atomic Ratio
Green fibers	32.94	44.37	6.80	1.21	12.66	2.02	2.96
Fibers in Ar (1000 °C)	43.82	39.51	14.88	1.89	/	/	1.90
Fibers in H_2_ (1000 °C)	51.95	28.32	17.10	2.63	/	/	1.15
Ar–Ar process fiber at 1600 °C	45.19	38.82	14.93	1.16	/	/	1.82
H_2_–Ar process fiber at 1600 °C	52.73	27.68	17.45	2.10	/	/	1.11
